# Supplementary material for: Enhanced processivity and collective force production of kinesin-1 at low radial forces
Source: eLife. 2026 Jul 20;14:RP109012. doi: 10.7554/eLife.109012 (PMC13384491; doi:10.7554/eLife.109012)

Figure 1_figure supplement 1A

K560 GFP Biotin

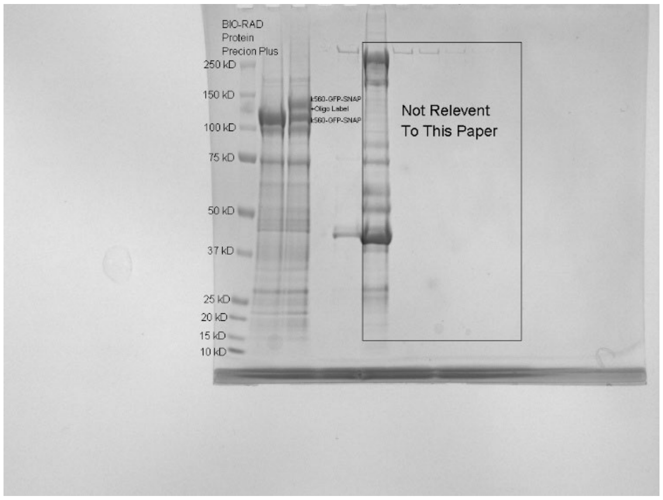

KIF5B-GFP-Oligo

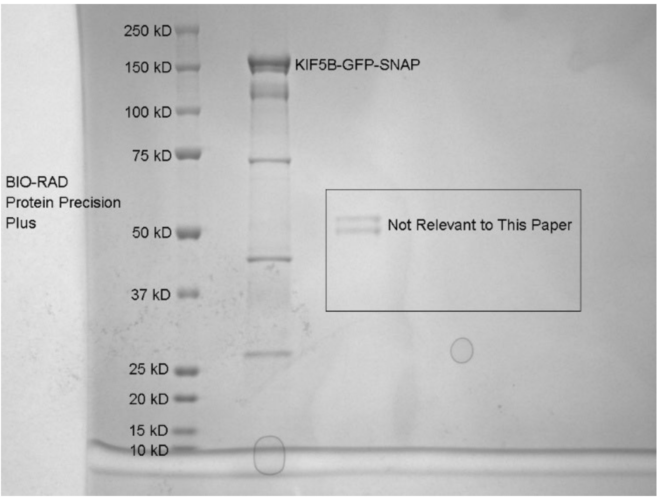

Figure 1_figure supplement 1B

DNA Handle Cy3

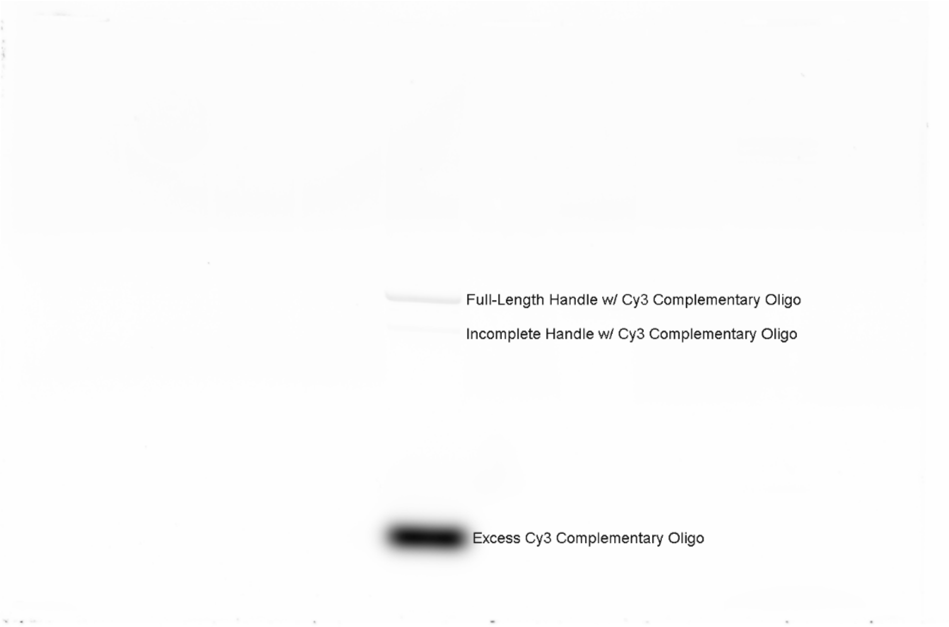

DNA Handle UV

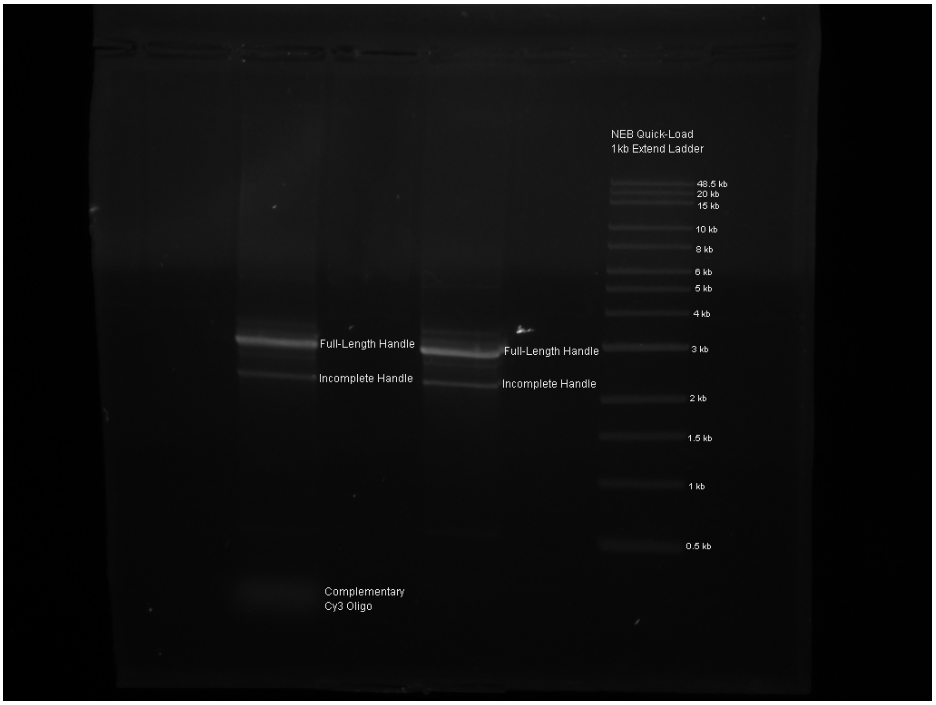

Supplement: Figure 1—figure supplement 1—source data 1. [file elife-109012-fig1-figsupp1-data1.pdf]
