# Supplementary figures and images for: Enhanced processivity and collective force production of kinesin-1 at low radial forces
Source: eLife. 2026 Jul 20;14:RP109012. doi: 10.7554/eLife.109012 (PMC13384491; doi:10.7554/eLife.109012)

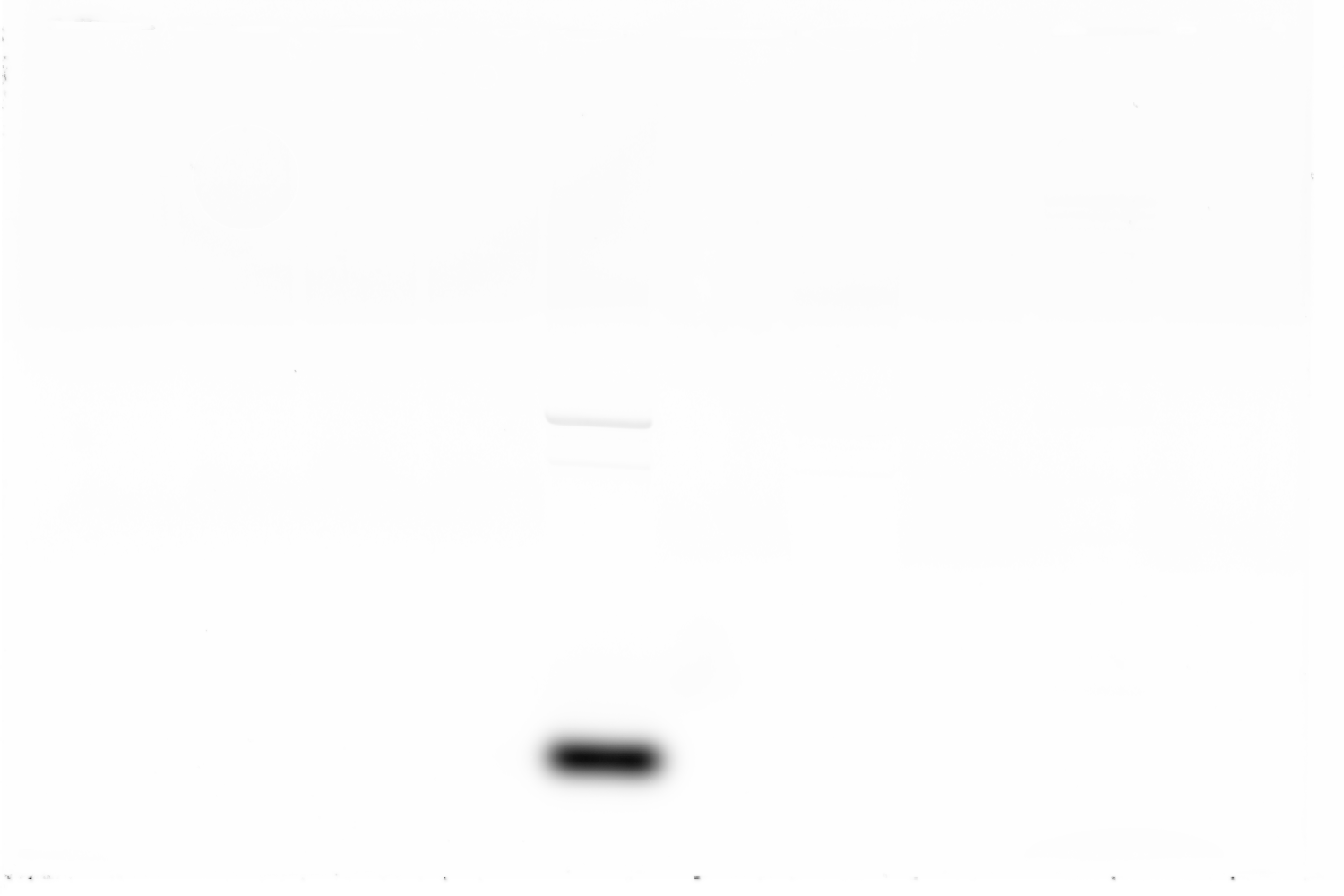

Supplement: Figure 1—figure supplement 1—source data 2. [file elife-109012-fig1-figsupp1-data2.zip › A,B - Raw Gels/Handle + Cy3 Oligo Typhoon.tif]

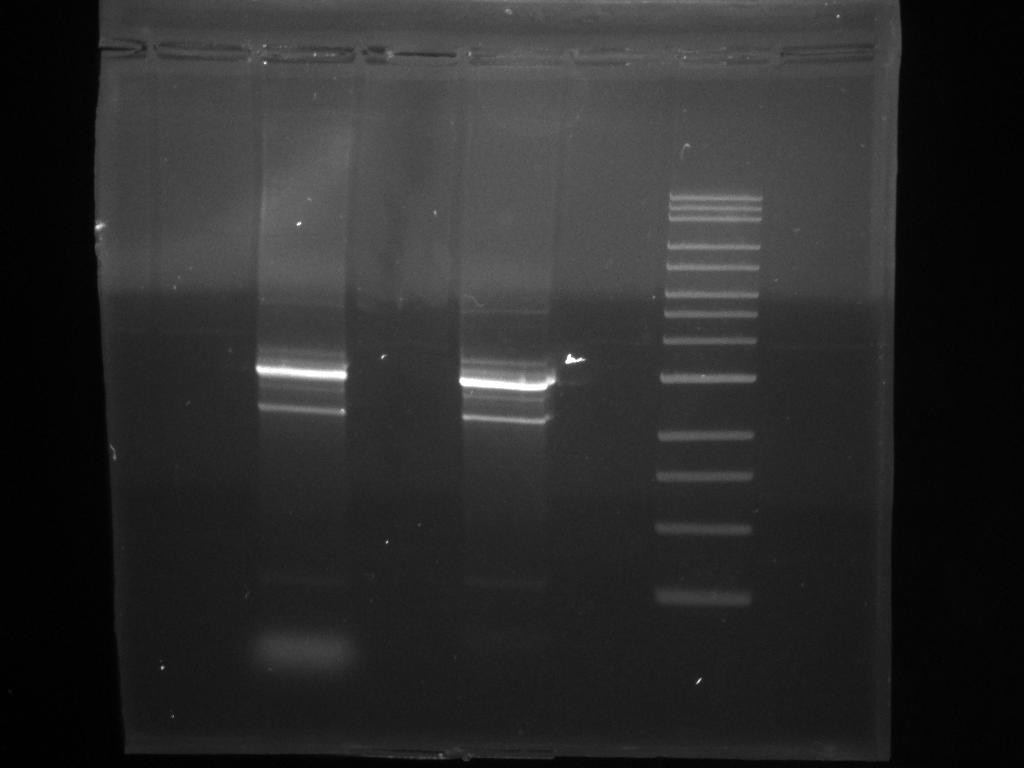

Supplement: Figure 1—figure supplement 1—source data 2. [file elife-109012-fig1-figsupp1-data2.zip › A,B - Raw Gels/Handle_+ Cy3 Oligo GelRed Stained and UV imaged.tif]

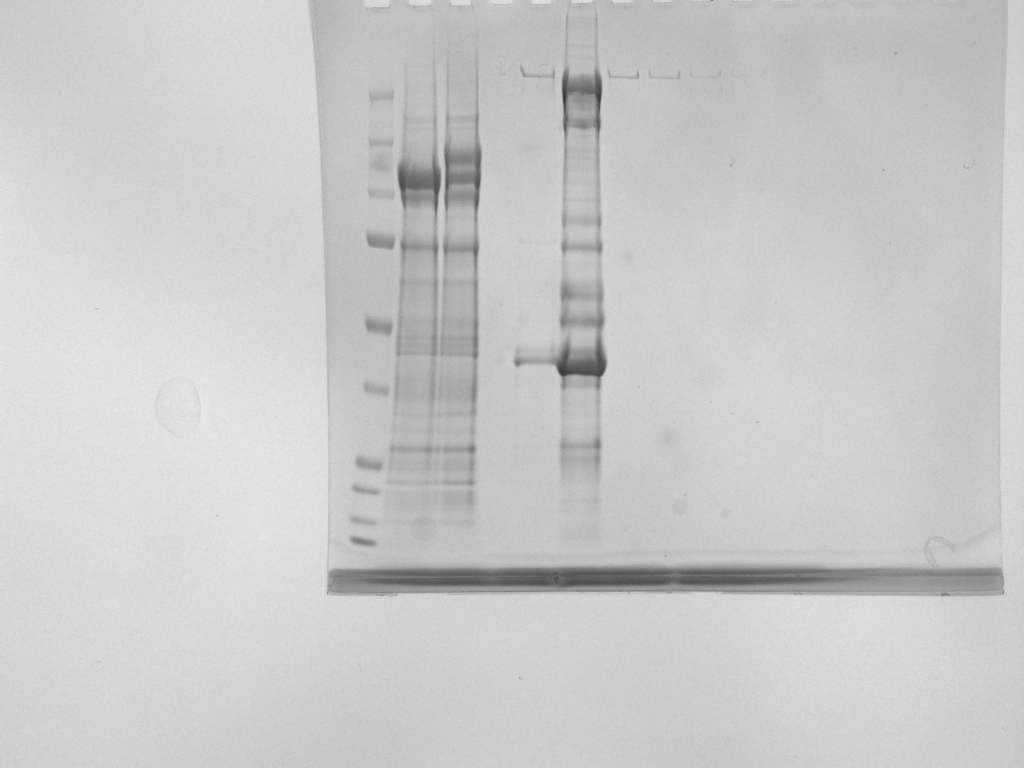

Supplement: Figure 1—figure supplement 1—source data 2. [file elife-109012-fig1-figsupp1-data2.zip › A,B - Raw Gels/K560-GFP-Biotin_raw.tif]

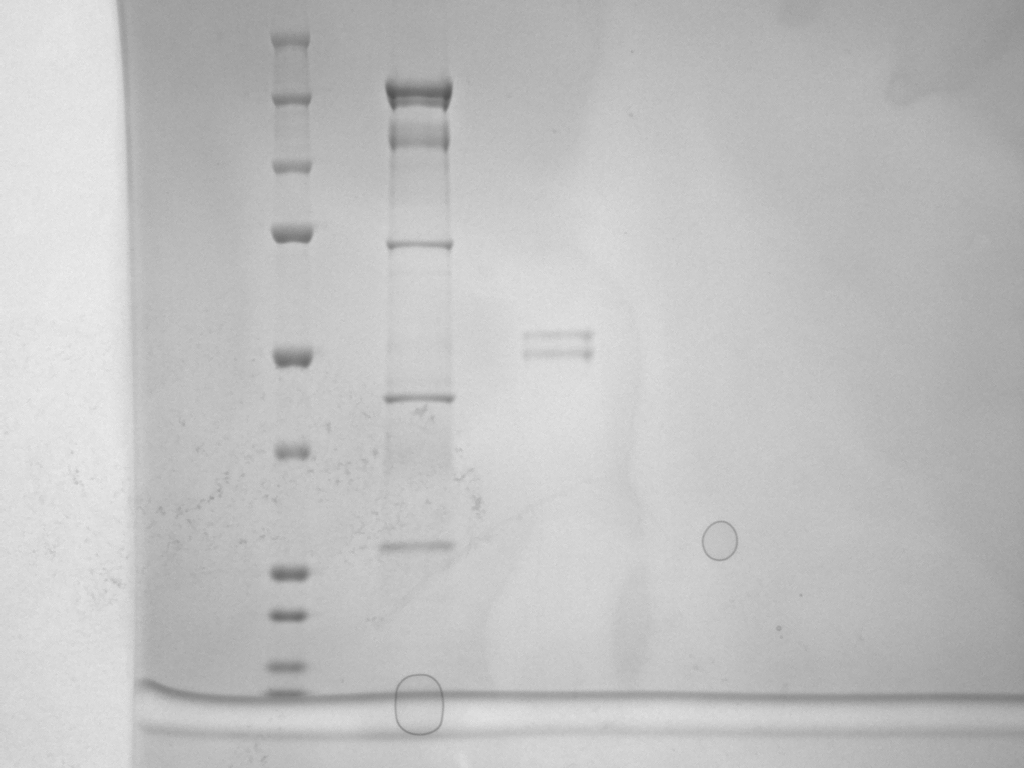

Supplement: Figure 1—figure supplement 1—source data 2. [file elife-109012-fig1-figsupp1-data2.zip › A,B - Raw Gels/KIF5B_GFP_Oligo_raw.tif]

Figure 3\_figure supplement 1A

K560 GFP Biotin

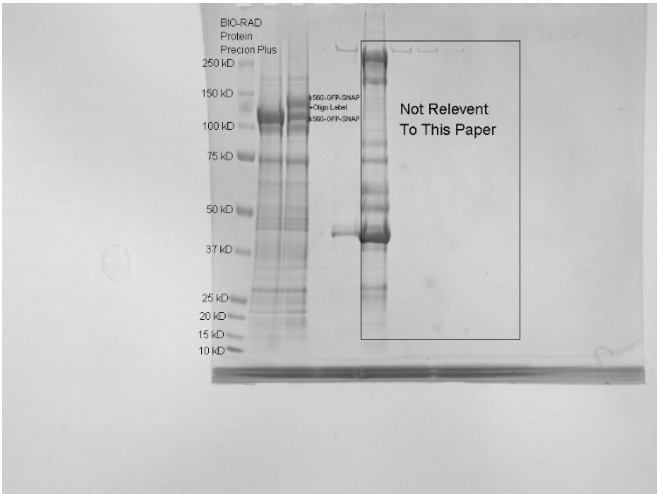

Chassis Gel

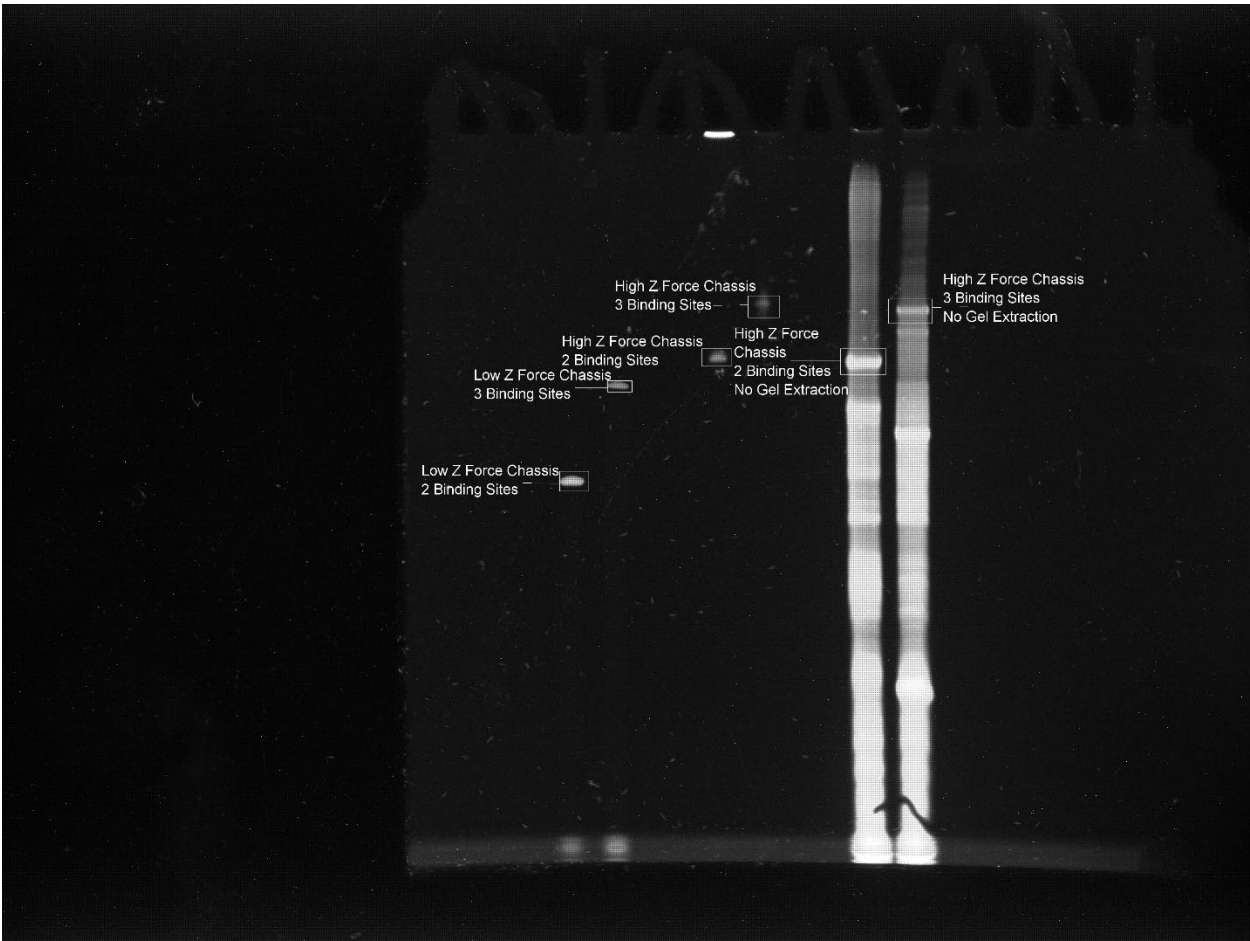

Supplement: Figure 3—figure supplement 1—source data 1. [file elife-109012-fig3-figsupp1-data1.pdf]

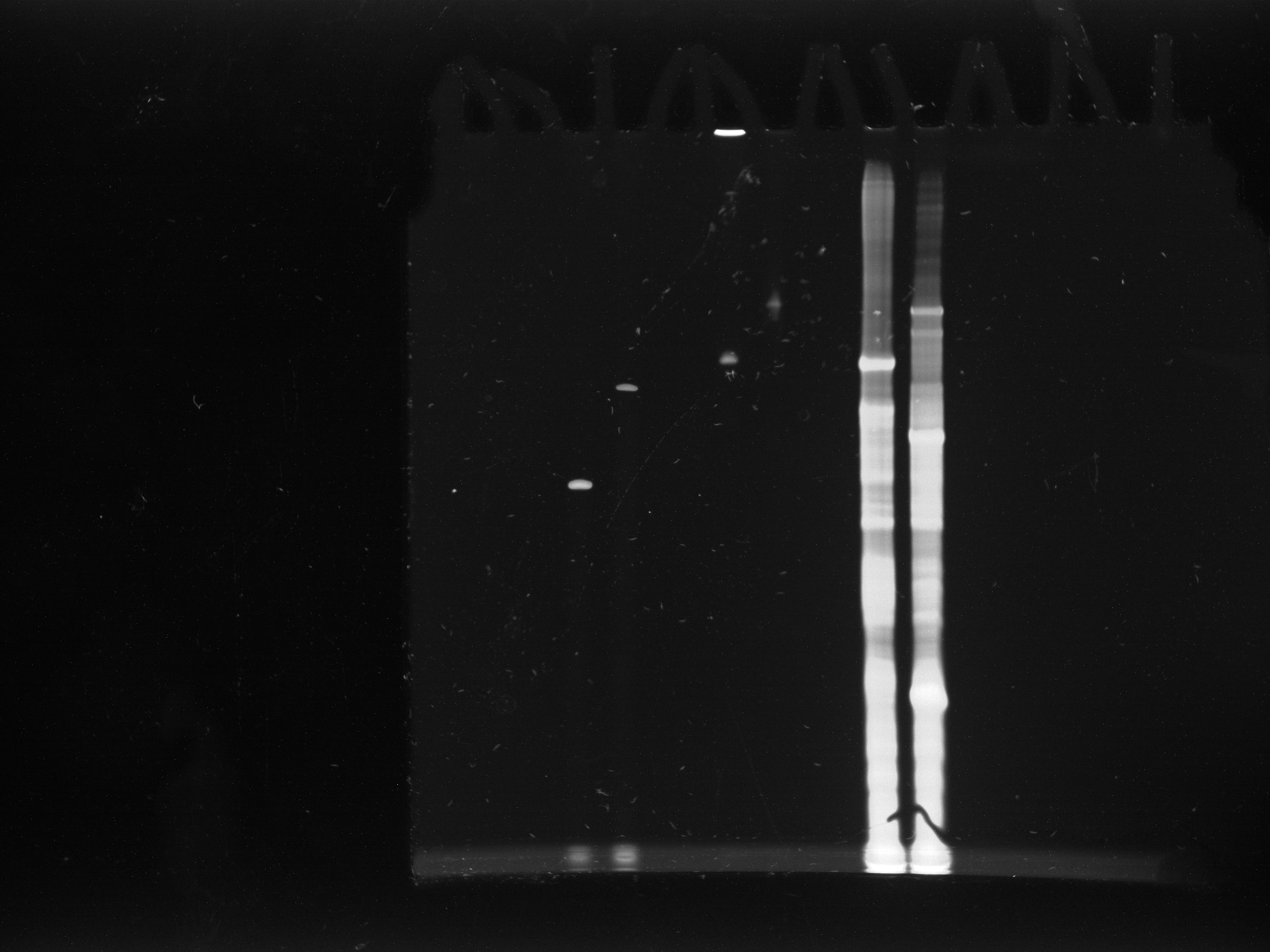

Supplement: Figure 3—figure supplement 1—source data 2. [file elife-109012-fig3-figsupp1-data2.zip › Raw Gels/Chassis_Gel.tiff]
