# Supplementary material for: Enhanced processivity and collective force production of kinesin-1 at low radial forces
Source: eLife. 2026 Jul 20;14:RP109012. doi: 10.7554/eLife.109012 (PMC13384491; doi:10.7554/eLife.109012)
Supplement: Peer review history

# Editor report: eLife Assessment

**DOI:** 10.7554/eLife.109012.3.sa0

**Authors:** Kerry Bloom

**Affiliations:** The University of North Carolina at Chapel Hill United States

**Keywords:** Compelling, Important

The current work uses DNA-tethered motor trapping to reduce vertical forces and improve datasets for kinesin-1 motility under load. The evidence is **compelling** and the significance is **important** to the kinesin field. Kinesin-1 is more robust and less prone to premature detachment than previously indicated. This represents a significant advancement in the field and is generally applicable to work with optical tweezers.

# Referee report: Reviewer #1 (Public review)

**DOI:** 10.7554/eLife.109012.3.sa1

Summary:

The manuscript by Hensley and Yildez studies the mechanical behavior of kinesin under conditions where the z-component of the applied force is minimized. This is accomplished by tethering the kinesin to the trapped bead with a long double stranded DNA segment as opposed to directly binding the kinesin to the large bead. It complements several recent studies that have used different approaches to looking at the mechanical properties of kinesin under low z-force loads. The study shows that much of the mechanical information gleaned from the traditional "one bead" with attached kinesin approach was probably profoundly influenced by the direction of the applied force. The authors speculate that when moving small vesicle cargos (particularly membrane bound ones) the direction of resisting force on the motor has much less of a z-component than might be experience if the motor were moving large organelles like mitochondria.

Strengths:

The approach is sound and provides an alternative method to examine the mechanics of kinesin under conditions where the z-component of the force is lessened. The data show that kinesin has very different mechanical properties compared to those extensively reported with using the "single-bead" assay where the molecule is directly coupled to a large bead which is then trapped.

Weaknesses:

The sub stoichiometry binding of kinesins to the multivalent DNA complicates the interpretation of the data.

Comments on revisions:

The authors have addressed my concerns.

# Referee report: Reviewer #2 (Public review)

**DOI:** 10.7554/eLife.109012.3.sa2

This short report by Hensley and Yildiz explores kinesin-1 motility under more physiological load geometries than previous studies. Large Z-direction (or radial) forces are a consequence of certain optical trap experimental geometries, and likely do not occur in the cell. Use of a long DNA tether between the motor and the bead can alleviate Z-component forces. The authors perform three experiments. In the first, they use two assay geometries - one with kinesin attached directly to a bead and the other with kinesin attached via a 2 kbp DNA tether - with a constant-position trap to determine that reducing the Z component of force leads to a difference in stall time but not stall force. In the second, they use the same two assay geometries with a constant-force trap to replicate the asymmetric slip bond of kinesin-1; reducing the Z component of force leads to a small but uniform change in the run lengths and detachment rates under hindering forces but not assisting forces. In the third, they connect two or three kinesin molecules to each DNA, and measure a stronger scaling in stall force and time when the Z component of force is reduced. They conclude that kinesin-1 is a more robust motor than previously envisaged, where much of its weakness came from the application of axial force. If forces are instead along the direction of transport, kinesin can hold on longer and work well in teams. The experiments are rigorous, and the data quality is very high. There is little to critique or discuss. The improved dataset will be useful for modeling and understanding multi-motor transport. The conclusions complement other recent works that used different approaches to low-Z component kinesin force spectroscopy, and provide strong value to the kinesin field.

Comments on revisions:

The authors have satisfied all of my comments. I commend them on an excellent paper.

# Referee report: Reviewer #3 (Public review)

**DOI:** 10.7554/eLife.109012.3.sa3

Hensley et al. present an important study into the force-detachment behaviour of kinesin-1, using a newly adapted methodological approach. This new method of DNA-tethered motor trapping is effective in reducing vertical forces and can be easily optimised for other motors and protein characterisation. The major strength of the paper is characterising kinesin-1 under low z-forces, which is likely to reflect the physiological scenario. They find kinesin-1 is more robust and less prone to premature detachment. The motors exhibit higher stall rates and times. Under hindering and assisting loads, kinesin-1 detachment is more asymmetric and sensitive, and with low z-force shows that slip-behaviour kinetics prevail. Another achievement of this paper is the demonstration of the multi-motor kinesin-1 assay using their low-z force method, showing that multiple kinesin-1 motors are capable of generating higher forces (up to 15 pN, and nearly proportional to motor number), thus opening an avenue to study multiple motor coordination. Overall, the data have been collected in a rigorous manner, the new technique is sound and effective, and results presented are compelling.

# Author comment: Author response

**DOI:** 10.7554/eLife.109012.3.sa4

**Authors:** Andrew M Hensley, Ahmet Yildiz

**Affiliations:** https://ror.org/01an7q238 University of California, Berkeley Berkeley United States; https://ror.org/01an7q238 University of California, Berkeley Berkeley United States

The following is the authors’ response to the original reviews

**Reviewer #1 (Recommendations for the authors):**

(1) My primary concern is that in some of the studies, there are not enough data points to be totally convincing. This is particularly apparent in the low z-force condition of Figure 1C.

We agree that adequate sampling is essential for drawing robust conclusions. To address this concern, we performed a post hoc sensitivity analysis to assess the statistical power of our dataset. Given our sample sizes (N = 85 and 45) and observed variability, the experiment had 80% power (α = 0.05) to detect a difference in stall force of approximately 0.36 pN (Cohen’s d ≈ 0.38). The actual difference observed between conditions was 0.25 pN (d ≈ 0.26), which lies below the minimum detectable effect size. Thus, the non-significant result (p = 0.16) likely reflects that any true difference, if present, is smaller than the experimental sensitivity, rather than a lack of sufficient sampling.

Importantly, both measured stall forces fall within the reported range for kinesin-1 in the literature, supporting that the dataset is representative and the measurements are reliable.

(2) I'm also concerned about Figure 2B. Does each data point in the three graphs represent only a single event? If so, this should probably be repeated several more times to ensure that the data are robust.

Each data point shown corresponds to the average of many processive runs, ranging from 32 to 167. This has been updated in the figure caption accordingly.

(3) Figure 3. I'm surprised that the authors could not obtain a higher occupancy of the multivalent DNA tether with kinesin motors. They were adding up to a 30X higher concentration of kinesin, but still did not achieve stoichiometric labeling. The reasons for this should be discussed. This makes interpretation of the mechanical data much tougher. For instance, only 6-7% of the beads would be driven by three kinesins. Unless the movement of hundreds of beads were studied, I think it would be difficult to draw any meaningful insight, since most of the events would be reflective of beads with only one or sometimes two kinesins bound. I think more discussion is required to describe how these data were treated.

The mass-photometry data in Figure 3B were acquired in the presence of a 3-fold molar excess of kinesin (Supplemental Figure 4) relative to the DNA chassis. In comparison, optical trapping studies were performed at a 10-20-fold molar excess of kinesin, resulting in a substantially higher percentage of chassis with multiple motors. The reason why we had to perform mass photometry measurements at lower molar excess than the optical trap is that at higher kinesin concentrations, the “kinesin-only” peak dominated and obscured 2- or 3-kinesin-bound species, preventing reliable fitting of the mass photometry data.

We have now used the mass photometry measurements to extrapolate occupancies under trapping conditions. We estimate 76-93% of 2-motor chassis are bound to two kinesins and ~70% of 3-motor chassis are bound to three kinesins under our trapping conditions. Moreover, the mean forces in Figures 3C–D exceed those expected for a single kinesin, consistent with occupancy substantially greater than one motor per chassis.

We wrote: “To estimate the percentage of chassis with two and three motors bound, we performed mass photometry measurements at a 3-fold molar excess of kinesin to the chassis, as higher ratios would obscure the distinction of complexes from the kinesin-only population. Assuming there is no cooperativity among the binding sites, we modeled motor occupancy using a Binomial distribution (Figure 3_figure supplement 2). We observed 17-29% of particles corresponded to the two-motor species on the 2-motor chassis in mass photometry, indicating that 45-78% of the 2-motor chassis was bound to two kinesins. Similarly, 15% and 40% of the 3motor chassis were bound to two and three kinesins, respectively.

In optical trapping assays, we used 10-fold and 20-fold molar excess of kinesin for 2-motor and 3-motor chassis, respectively, to substantially increase the percentage of the chassis carried by multiple kinesins. Under these conditions, we estimate 76-93% of the 2-motor chassis were bound to two kinesins, and 30% and 70% of 3-motor chassis were bound to two and three kinesins, respectively.”

“Multi-motor trapping assays were performed similarly using 10x and 20x kinesin for 2- and 3motor chassis, respectively. To estimate the percentage of chassis with multiple motors, we used the probability of kinesin binding to a site on a chassis from mass photometry in 3x excess condition to compute an effective dissociation constant $\kappa=r \cdot \frac{1-p}{p}$ where *r* is the molar ratio of kinesin to chassis. Single-site occupancy at higher molar excesses of kinesin was calculated using this parameter. ”

We also added Figure 3_figure supplement 2 to explain our Binomial model.

(4) Page 5, 1st paragraph. Here, the authors are comparing time constants from stall experiments to data obtained with dynein from Ezber et al. This study used the traditional "one bead" trapping approach with dynein bound directly to the bead under conditions where it would experience high z-forces. Thus, the comparison between the behavior of kinesin at low z-forces is not necessarily appropriate. Has anyone studied dynein's mechanics under low z-force regimes?

We thank the reviewer for catching a citation error. The text has been corrected to reference Elshenawy et al. 2020, which reported stall time constants for mammalian dynein.

To our knowledge, dynein’s mechanics under explicitly low z-force conditions have not yet been reported; however, given the more robust stalling behavior of dynein and greater collective force generation, the cited paper was chosen to compare low z-force kinesin to a motor that appears comparatively unencumbered by z-forces. Our study adds to growing evidence that high z-forces disproportionately limit kinesin performance.

For clarification, we modified that sentence as follows: “These time constants are comparable to those reported for minus-end-directed dynein under high z-forces”.

**Reviewer #2 (Recommendations for the authors):**

(1) P3 pp2, a DNA tensiometer cannot control the force, but it can measure it; get the distance between the two ends of the tensiometer, and apply WLC.

The text has been updated to more accurately reflect the differences between optical trapping and kinesin motility against a DNA tensiometer with a fixed lattice position.

(2) Fig. 2b, SEM is a poor estimate or error for exponentially distributed run lengths. Other methods, like bootstrapping an exponential distribution fit, may provide a more realistic estimate.

Run lengths were plotted as an inverse cumulative distribution function and fitted to a single exponential decay (Supplementary Figure S3). The plotted value represents the fitted decay constant (characteristic run length) ± SE (standard error of the fit), not the arithmetic mean ± SEM. Velocity values are reported as mean ± SEM. Detachment rate was computed as velocity divided by run length, except at 6 and 10 pN hindering loads, where minimal forward displacement necessitated fitting run-time decays directly. In those cases, the plotted detachment rate equals the inverse of the fitted time constant. The figure caption has been updated accordingly.

(3) Kinesin-1 is covalently bound to a DNA oligo, which then attaches to the DNA chassis by hybridization. This oligo is 21 nt with a relatively low GC%. At what force does this oligo unhybridize? Can the authors verify that their stall force measurements are not cut short by the oligo detaching from the chassis?

The 21-nt attachment oligo (38 % GC) is predicted to have ΔG_37C_ ≈-25 kcal/mole or approximately 42 kT. If we assume this is the approximate amount of work required to unhybridize the oligo, we would expect the rupture force to be >15 pN. This significantly exceeds the stall force of a single kinesin. Since the stalling events rarely exceed a few seconds, it is unlikely that our oligos quickly detach from the chassis under such low forces.

Furthermore, optical trapping experiments are tuned such that no more than 30% of beads display motion within several minutes after they are brought near microtubules. After stalling events, the motor dissociates from the MT, and the bead snaps back to the trap center. Most beads robustly reengage with the microtubule, typically within 10 s, suggesting that the same motor chassis reengages with the microtubule after microtubule detachment. Successive runs of the same bead typically have similar stall forces, suggesting that the motors do not disengage from the chassis under resistive forces exerted by the trap.

(4) Figure 1, a justification or explanation should be provided for why events lower than 1.5 pN were excluded. It appears arbitrary.

Single-motor stall-force measurements used a trap stiffness of 0.08–0.10 pN/nm. At this stiffness, a 1.5 pN force corresponds to 15–19 nm bead displacement, roughly two kinesin steps, and events below this threshold could not be reliably distinguished from Brownian noise. For this reason, forces < 1.5 pN were excluded.

In Methods, we wrote “Only peak forces above 1.5 pN (corresponding to a 15-19 nm bead displacement) were analyzed to clearly distinguish runs from the tracking noise.”

(5) Figure 2b, is the difference in velocity statistically significant?

The difference in velocity is statistically significant for most conditions. We did not compare velocities for -10 and -6 pN as these conditions resulted in little forward displacement. However, the p-values for all of the other conditions are -4 pN: 0.0026, -2 pN: 0.0001, -1 pN: 0.0446, +0.5 pN: 0.3148, +2 pN: 0.0001, +3 pN: 0.1191, +4 pN: 0.0004.

(6) The number of measurements for each experimental datapoint in the corresponding figure caption should be provided. SEM is used without, but N is not reported in the caption.

Figure captions have now been updated to report the number of trajectories (N) for each data point.

**Reviewer #3 (Recommendations for the authors):**

(1) The method of DNA-tethered motor trapping to enable low z-force is not entirely novel, but adapted from Urbanska (2021) for use in conventional optical trapping laboratories without reliance on microfluidics. However, I appreciate that they have fully established it here to share with the community. The authors could strengthen their methods section by being transparent about protein weight, protein labelling, and DNA ladders shown in the supplementary information. What organism is the protein from? Presumably human, but this should be specified in the methods. While the figures show beautiful data and exemplary traces, the total number of molecules analysed or events is not consistently reported. Overall, certain methodological details should be made sufficient for reproducibility.

We appreciate the reviewer’s attention to methodological clarity. The constructs used are indeed human kinesin-1, KIF5B. The Methods now specify protein origin, molecular weights, and labeling details, and all figure captions report the number of trajectories analyzed to ensure reproducibility.

(2) The major limitation the study presents is overarching generalisability, starting with the title. I recommend that the title be specific to kinesin-1.

The title has been revised to specify kinesin-1.

The study uses two constructs: a truncated K560 for conventional high-force assays, and full-length Kif5b for the low z-force method. However, for the multi-motor assay, the authors use K560 with the rationale of preventing autoinhibition due to binding with DNA, but that would also have limited characterisation in the single-molecule assay. Overall, the data generated are clear, high-quality, and exciting in the low z-force conditions. But why have they not compared or validated their findings with the truncated construct K560? This is especially important in the force-feedback experiments and in comparison with Andreasson et al. and Carter et al., who use Drosophila kinesin-1. Could kinesin-1 across organisms exhibit different force-detachment kinetics? It is quite possible.

Construct choice was guided by physiological relevance and considerations of autoinhibition: K560 was used for high z-force single-motor assays. The results of these assays are consistent with conventional bead assays performed by Andreasson et al. and Carter et al. using kinesin from a different organism. Therefore, we do not believe there are major differences between force properties of Drosophila and human kinesin-1.

For low z-force assays, we used full-length KIF5B, which has nearly identical velocity and stall force to K560 in standard bead assays. We used this construct for low z force assays because it has a longer and more flexible stalk than K560 and better represents the force behavior of kinesin under physiological conditions. We then used constitutively-active K560 motors for multi-motor experiments to avoid potential complications from autoinhibition of full-length kinesin.

Similarly, the authors test backward slipping of Kif5b and K560 and measure dwell times in multi-motor assays. Why not detail the backward slippage kinetics of Kif5b and any step-size impact under low z-forces? For instance, with the traces they already have, the authors could determine slip times, distances, and frequency in horizontal force experiments. Overall, the manuscript could be strengthened by analysing both constructs more fully.

Slip or backstep analyses were not performed on single-motor data because such events were rare; kinesin typically detached rather than slipped. In contrast, multi-motor assays exhibited frequent slip events corresponding to the detachment of individual motors, which were analyzed in detail.

We wrote “In comparison, slipping events were rarely observed in beads driven by a single motor, suggesting that kinesin typically detaches rather than slipping back on the microtubule under hindering loads.”

Appraisal and impact:

This study contributes to important and debated evidence on kinesin-1 force-detachment kinetics. The authors conclude that kinesin-1 exhibits a slip-bond interaction with the microtubule under increasing forces, while other recent studies (Noell et al. and Kuo et al.), which also use low z-force setups, conclude catch-bond behaviour under hindering loads. I find the results not fully aligned with their interpretation. The first comparison of low zforces in their setup with Noell et al. (2024), based on stall times, does not hold, because it is an apples-to-oranges comparison. Their data show a stall time constant of 2.52 s, which is comparable to the 3 s reported by Noell et al., but the comparison is made with a weighted average of 1.49 s. The authors do report that detachment rates are lower in low z-force conditions under unloaded scenarios. So, to completely rule out catch-bond-like behaviour is unfair. That said, their data quality is good and does show that higher hindering forces lead to higher detachment rates. However, on closer inspection, the range of 0-5 pN shows either a decrease or no change in detachment rate, which suggests that under a hindering force threshold, catch-bond-like or ideal-bond-like behaviour is possible, followed by slipbond behaviour, which is amazing resolution. Under assisting loads, the slip-bond character is consistent, as expected. Overall, the study contributes to an important discussion in the biophysical community and is needed, but requires cautious framing, particularly without evidence of motor trapping in a high microtubule-affinity state rather than genuine bond strengthening.

We are not completely ruling out the catch bond behavior in our manuscript. As the reviewer pointed out, our results are consistent with the asymmetric slip bond model, whereas DNA tensiometer assays are more consistent with the catch bond behavior. The advantage of our approach is the capability to directly control the magnitude and direction of load exerted on the motor in the horizontal axis and measure the rate at which the motor detaches from the microtubule as it walks under constant load. In comparison, DNA tensiometer assays cannot control the force, but measure the time it takes the motor to fall off from the microtubule after a brief stall. The extension of the DNA tether is used to estimate the force exerted on the motor during a stall in those assays. The slight disadvantage of our method is the presence of low zforces, whereas DNA tensiometer assays are expected to have little to no z-force. We wrote that the discrepancy between our results can be attributed to the presence of low z forces in our DNA tethered trapping assembly, which may result in a higher-than-normal detachment rate under high hindering loads, thereby resulting in less asymmetry in the force detachment kinetics. We also added that this discrepancy can be addressed by future studies that directly control and measure horizontal force and measure the motor detachment rate in the absence of z forces. Optical trapping assays with small nanoparticles (Sudhakar et al. Science 2021) may be well suited to conclusively reveal the bond characteristics of kinesin under hindering loads.

**Reviewing Editor Comments:**

The reviewers are in agreement with the importance of the findings and the quality of the results. The use of the DNA tether reduces the z-force on the motor and provides biologically relevant insight into the behavior of the motor under load. The reviewers' suggestions are constructive and focus on bolstering some of the data points and clarifying some of the methodological approaches. My major suggestion would be to clarify the rationale for concluding that kinesin-1 exhibits slip-bond behavior with increasing force in light of the work of Noell (10.1101/2024.12.03.626575) and Kuo et al (2022 10.1038/s41467022-31069-x), both of which take advantage of DNA tethers.

Please see our response to the previous comment. In the revised manuscript, we first clarified that our results are in agreement with previous theoretical (Khataee & Howard, 2019) and experimental studies (Kuo et al., 2022; Noell et al., 2024; Pyrpassopoulos et al., 2020) that kinesin exhibits slower detachment under hindering load. This asymmetry became clear when the z-force was reduced or eliminated.

We clarified the differences between our results and DNA tensiometer assays and provided a potential explanation for these discrepancies. We also proposed that future studies might be required to fully distinguish between asymmetric slip, ideal, or catch bonding of kinesin under hindering loads.

We wrote:

“Our results agree with the theoretical prediction that kinesin exhibits higher asymmetry in force-detachment kinetics without z-forces (Khataee & Howard, 2019), and are consistent with optical trapping and DNA tensiometer assays that reported more persistent stalling of kinesin in the absence of z-forces (Kuo et al., 2022; Noell et al., 2024; Pyrpassopoulos et al., 2020).

Force-detachment kinetics of protein-protein interactions have been modeled as either a slip, ideal, or catch bond, which exhibit an increase, no change, or a decrease in detachment rate, respectively, under increasing force (Thomas et al., 2008). Slip bonds are most commonly observed in biomolecules, but studies on cell adhesion proteins reported a catch bond behavior (Marshall et al., 2003). Although previous trapping studies of kinesin reported a slip bond behavior (Andreasson et al., 2015; Carter & Cross, 2005), recent DNA tensiometer studies that eliminated the z-force showed that the detachment rate of the motor under hindering forces is lower than that of an unloaded motor walking on the microtubule (Kuo et al., 2022; Noell et al., 2024), consistent with the catch bond behavior. Unlike these reports, we observed that the stall duration of kinesin is shorter than the motor run time under unloaded conditions, and the detachment rate of kinesin increases with the magnitude of the hindering force. Therefore, our results are more consistent with the asymmetric slip bond behavior. The difference between our results and the DNA tensiometer assays (Kuo et al., 2022; Noell et al., 2024) can be attributed to the presence of low z-forces in our DNA-tethered optical trapping assays, which may increase the detachment rate under high hindering forces. Future studies that could directly control hindering forces and measure the motor detachment rate in the absence of z-forces would be required to conclusively reveal the bond characteristics of kinesin under hindering loads.”
